# Exercise-derived extracellular vesicles in oncology: a new frontier for translational nanomedicine

**DOI:** 10.1186/s12967-026-07742-w

**Published:** 2026-01-28

**Authors:** Monica Silvestri, Cristina Fantini, Guglielmo Duranti, Elisa Grazioli, Daniela Caporossi, Carolina Balbi, Ivan Dimauro

**Affiliations:** 1 Unit of Biology and Genetics of Movement, Department of Movement, Human and Health Sciences, University of Rome Foro Italico, Piazza Lauro De Bosis 6, Rome, 00135 Italy; 2 Unit of Biochemistry and Molecular Biology, Department of Movement, Human and Health Sciences, University of Rome Foro Italico, Piazza Lauro De Bosis 6, Rome, 00135 Italy; 3 Unit of Physical Exercise and Sport Sciences, Department of Movement, Human and Health Sciences, University of Rome Foro Italico, Piazza Lauro De Bosis 6, Rome, 00135 Italy; 4 Center for Molecular Cardiology, University of Zurich, Schlieren, Switzerland; 5 Department of Medicine, Baden Cantonal Hospital, Baden, Switzerland

**Keywords:** Exercise-derived extracellular vesicles, Oncology, Nanomedicine, Cancer immunotherapy, Translational research

## Abstract

**Background:**

Exercise-derived extracellular vesicles (ExEVs) have emerged as pivotal mediators of the systemic benefits associated with physical activity (PA). Growing evidence highlights their involvement in modulating cancer biology, enhancing immune surveillance, and alleviating treatment-related toxicity.

**Main body:**

Recent preclinical studies have begun to unravel the molecular composition and functional characteristics of ExEVs, suggesting considerable potential as both biomarkers and therapeutic agents in oncology and cardio-oncology. There is also increasing interest in the development of extracellular vesicle-inspired nanotherapeutics, with the goal of replicating the anticancer and cardioprotective benefits observed with PA in patients with cancer. While these approaches show promise, significant translational challenges persist, including heterogeneity among vesicle populations, manufacturing complexities, and unresolved regulatory questions.

**Conclusions:**

To address these barriers, a coordinated strategy is necessary. This should include the establishment of standardized protocols, promotion of interdisciplinary collaboration, and integration with personalized oncology frameworks. Progress in the therapeutic application of ExEVs may ultimately facilitate the development of innovative, low-toxicity interventions for cancer care.

## Introduction

Over the past decade, physical activity (PA) has been shown to be an effective systemic anticancer strategy. To date, PAs are known to potentially affect several biological processes involved in tumor development and progression, such as inflammation, immunological surveillance, and metabolic regulation, in addition to improving quality of life and reducing adverse treatment effects [1–8].

This growing knowledge has inspired considerable interest in the development of “exercise mimetics” which are pharmacological agents designed to reproduce the complex molecular and systemic responses induced by PA [9-11]. These compounds offer a promising opportunity to deliver the anticancer and health-promoting effects of exercise in cancer patients, including those for whom regular PA is impractical or impossible owing to illness severity, treatment burden, or physical limitations. Current candidate molecules include 5’ adenosine monophosphate-activated protein kinase (AMPK) activators (via AICAR or other activators), peroxisome proliferator-activated receptor delta (PPARδ) agonists (with agonists such as GW1516), and agents capable of simulating muscle contraction signaling or inducing extracellular vesicle (EV) biogenesis [12–15]. Although many of these molecules are still in the preclinical or early clinical evaluation stages, the conceptual framework underlying their development represents a considerable paradigm shift, recognizing the potential of pharmacological strategies as adjuvants or substitutes for lifestyle interventions in cancer treatment.

A particularly significant breakthrough in this context is the identification of EVs as one of the key mediators of the systemic benefits of exercise [16]. These nanosized vesicles function as sophisticated intercellular messengers, facilitating the transfer of proteins, lipids, metabolites, mRNAs, a range of noncoding RNAs, and even DNA fragments between cells [17]. This dynamic exchange has the capacity to modulate the function and phenotype of recipient cells, thereby orchestrating complex physiological responses at the tissue and systemic levels.

In recent years, an expanding body of research has focused on the molecular composition and functional roles of EVs released into circulation during and following PA [18–20]. These EVs are influenced by intensity [21, 22], duration, and patient status [23, 24]. Recent evidence indicates that exercise triggers the release of EVs enriched with proteins, metabolites, and regulatory RNAs that act as systemic messengers between skeletal muscle and distant organs (e.g., adipose tissue and brain) [16, 19, 23, 25–27]. Several studies show that both acute and chronic exercise significantly modulate the miRNA cargo of circulating EVs, including key myomiRs involved in muscle remodeling [25], metabolic regulation [28], immune function [29], and stress responses [30, 31]. Resistance training, in particular, induces marked changes in EV-associated miRNAs, such as miR-1, miR-133a/b, miR-206, miR-221, and miR-149, which are implicated in hypertrophy, angiogenesis, and neuromuscular adaptation [25]. Similarly, Lisi and colleagues showed that aerobic exercise acutely and chronically modulates the redox-related cargo of circulating EVs [30, 31]. A single bout of moderate-intensity aerobic exercise alters EV-associated oxidative stress markers and stress-responsive proteins, with untrained individuals displaying a more oxidized EV profile (higher protein carbonyls, Catalase, SOD2, HSP27/70) compared to trained subjects [31]. Interestingly, five consecutive days of aerobic training reduce EV oxidative damage and normalize antioxidant and heat-shock protein levels, shifting EVs toward a more balanced redox phenotype [30]. To date, the results reported in the literature are still heterogeneous, with strong variability related to exercise modality, sampling times, and methodological differences in EV isolation. However, despite these limitations, exercise-derived EVs (ExEVs) are increasingly recognized as potent endocrine mediators that drive physiological adaptation.

In addition to their role in the systemic response to physiological stress (e.g., physical activity) and in musculoskeletal remodeling, ExEVs may contribute to the broader health benefits of PA, including cancer prevention [32]. Long-term physically active individuals and previously sedentary subjects undergoing structured exercise interventions display a shared downregulation of specific exosomal miRNAs, such as let-7 family members, miR-15a/b, miR-23a, miR-142, miR-150, miR-199a/b, and miR-223, that regulate pathways associated with tumor progression, metabolism, inflammation, and cellular stress [32]. These findings suggest that ExEV-mediated signaling may influence tumor biology by modulating proliferative and survival pathways, supporting the emerging hypothesis that exercise-induced vesicular communication contributes to the anticancer effects of physical activity. Together, these data underscore the relevance of ExEVs as mechanistic mediators linking exercise to systemic adaptations, including those with potential therapeutic significance in oncology.

Mechanistically, exercise elicits a coordinated systemic response that promotes the release of EVs from diverse tissue sources, including skeletal muscle, immune cells, and adipose tissue [16, 19, 21, 33]. These vesicles act as carriers of complex molecular cargo, and emerging evidence indicates their capacity to remodel the tumor microenvironment (TME): they attenuate proinflammatory cytokine levels, improve the infiltration and activity of cytotoxic immune cells, and reprogram cancer cell metabolism. Additionally, ExEVs may play a role in normalizing the tumor vasculature, potentially improving both drug delivery and tissue oxygenation, factors critically linked to the efficacy of chemotherapy and radiotherapy [37, 38]. This line of research highlights the potential for ExEVs not only to act as biomarkers of the exercise response but also to serve as vehicles for targeted modulation of the tumor environment.

Based on these insights, the development of EV-inspired nanotherapeutics represents a promising next step in the evolution of cancer treatment strategies. Synthetic nanoparticles engineered to mimic the cargo and membrane composition of ExEVs are under active investigation for their capacity to enable targeted drug delivery and immune modulation within oncological contexts [39–41]. Leveraging in-depth knowledge of EV biology, these platforms aim to offer scalable, controllable, and regulatory-compliant alternatives to natural vesicles, potentially overcoming limitations of current delivery systems and enabling more precise therapeutic interventions. To provide a clear overview of the current therapeutic landscape, Table 1 summarizes the key similarities and differences between native exercise-derived EVs, engineered EVs, and exercise-inspired EV-mimetic nanoparticles in terms of their biological origin, functional properties, and translational potential in oncology.

**Table 1:** Comparison of native Exercise-Derived EVs (ExEVs), engineered EVs, and Exercise-Inspired EV-Mimetic nanoparticles

Category	Source/Production	Biological Features	Advantages	Limitations	Applications in Oncology
Native Exercise-Derived EVs (ExEVs)	Released naturally during or after exercise; derived from skeletal muscle, immune cells, endothelium, adipose tissue.	Complex, synergistic cargo (miRNAs, proteins, metabolites, myokines); shaped by exercise intensity, duration, and physiological status.	Physiologically relevant; low immunogenicity; reflect systemic exercise adaptations; ability to modulate TME.	Limited scalability; high donor variability; difficult GMP standardization; heterogeneous molecular composition.	Biomarkers of exercise adaptation; TME remodeling; chemo-/radio-sensitization; potential adjunct to standard therapies.
Engineered EVs	Isolated from donor cells and modified (drug loading, surface ligands, genetic engineering).	Tunable cargo and targeting ligands; semi-reproducible depending on parental cell line.	Precise targeting; customizable cargo; improved stability and biodistribution.	Complex, costly production; may alter natural EV functions; regulatory hurdles similar to biologics.	Targeted drug delivery; gene/miRNA therapy; immune modulation.
Exercise-Inspired EV-Mimetic Nanoparticles	Synthetic nanoparticles (e.g., LNPs) engineered to mimic ExEV membrane motifs and incorporate selected cargo.	Highly defined structure; incorporate selected “exercise-beneficial” molecules.	High scalability; reproducible manufacturing; controlled composition; strong regulatory compatibility.	Reduced biological complexity; may lack synergistic effects of natural ExEVs; possible off-target effects.	Precision oncology; reconstitution of exercise-derived signaling; cardioprotection; EV-inspired therapeutic platforms.

EV, Extracellular Vesicle; ExEVs, Exercise-derived EVs; GMP, Good Manufacturing Practice; LNPs, Lipid-nanoparticles; TME, Tumor microenvironment

Altogether, the convergence of exercise oncology, EV research, and advances in synthetic biology is poised to usher in a new era of personalized, non-invasive cancer interventions. Nevertheless, substantial challenges persist in translating these promising preclinical findings into clinical practice, particularly across the heterogeneous landscape of cancer types and patient populations. Further interdisciplinary research will be essential to refine these strategies, optimize their safety and efficacy, and thus realize their full potential in the context of modern cancer therapy.

Despite these advances, no comprehensive review has yet provided an integrated translational perspective linking the biological mechanisms of ExEVs to their potential clinical applications. This review addresses this critical gap by synthesizing the most recent evidence and proposing a strategic roadmap for their development as novel oncologic interventions.

## Therapeutic potential of EVs in oncology

Extracellular vesicles (EVs) have emerged as highly promising tools in cancer therapeutics. Several intrinsic features distinguish them from traditional drug delivery systems. Their biological compatibility and low immunogenicity are critical advantages. These properties minimize the risk of adverse immune reactions and improve suitability for clinical use [42–45]. EVs also display a remarkable natural ability to cross physiological barriers, such as the blood-brain barrier. This is typically challenging for synthetic nanoparticles or free drugs [46, 47]. As a result, EVs can achieve targeted delivery to specific tissues or organs, thereby maximizing therapeutic efficacy while minimizing systemic toxicity [33].

Another important property of EVs is their ability to encapsulate and transport a diverse array of molecular cargos, ranging from small-molecule chemotherapeutics to nucleic acids, proteins, and even CRISPR components [48, 49]. This versatility enables the development of customizable, targeted interventions tailored to individual tumor molecular profiles.

**Fig. 1:**
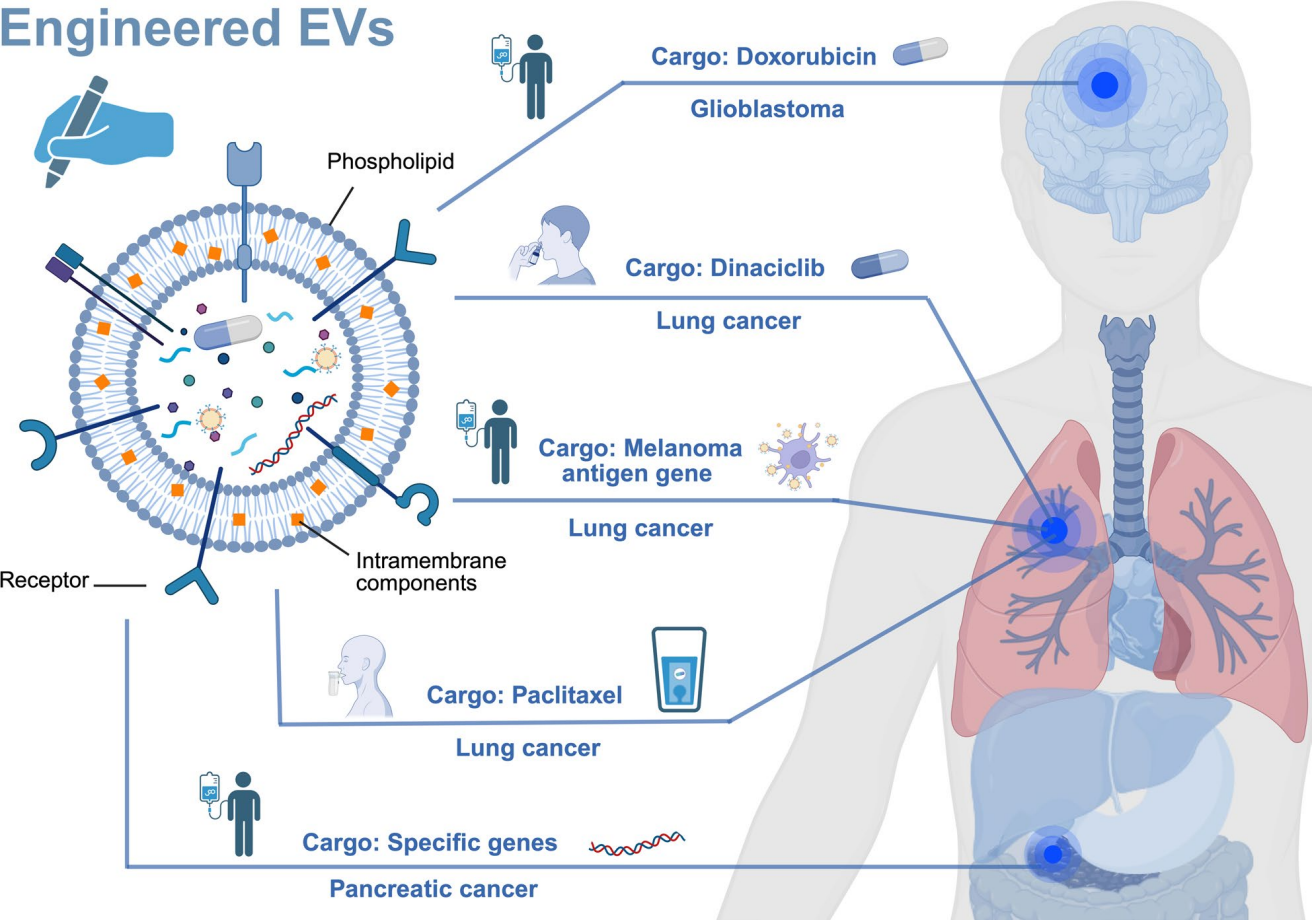
Engineered extracellular vesicles (EVs) in oncology. Schematic representation of the structural components of EVs (phospholipid bilayers, membrane proteins, and receptors) and their application as drug delivery platforms. Depending on their cargo, engineered EVs can be designed to transport chemotherapeutics, nucleic acids, or proteins. Examples include EVs loaded with doxorubicin for glioblastoma (intravenous delivery), dinaciclib-loaded EVs for lung cancer (aerosol delivery), dendritic cell–derived EVs carrying the melanoma antigen gene (MAGE) for NSCLC (immunotherapy), macrophage-derived EVs encapsulating paclitaxel for multidrug-resistant tumors, and EVs engineered to deliver therapeutic genes to KRAS-mutant pancreatic cancer. These approaches highlight the versatility of EVs in oncology, with multiple delivery routes (intravenous, inhaled, oral) being explored to maximize their efficacy and minimize off-target toxicity. EV, extracellular vesicle; NSCLC, non–small cell lung cancer; MAGE, melanoma antigen gene; KRAS, Kirsten rat sarcoma viral oncogene homolog

To address the well-documented limitations of conventional chemotherapy, including poor aqueous solubility, chemical instability, and nonspecific systemic distribution, engineered EVs have been developed as sophisticated drug delivery platforms (Fig. 1). For example, recent research highlights the use of EVs loaded with chemotherapeutic agents such as doxorubicin (Dox) for the treatment of glioblastoma [50] (Fig. 1). These engineered vesicles, which are typically administered intravenously, have demonstrated superior targeting of cancer cells, resulting in improved therapeutic efficacy and a reduction in off-target toxicity. These EVs not only improve drug bioavailability and selective uptake by cancer cells but also appear to extend the pharmacokinetic profile of the chemotherapeutic agent, potentially allowing for less frequent dosing and improved patient outcomes [50].

In this field, innovative approaches have also been developed to overcome the solubility and stability issues that plague some anticancer agents. An interesting example involves the encapsulation of dinaciclib in genetically modified EVs for the treatment of lung cancer (Fig. 1). Traditional formulations of dinaciclib exhibit poor solubility and rapid degradation; however, encapsulation within EVs has been shown to improve these issues. Specifically, aerosolized administration of these EVs has proven to be an effective alternative to intravenous injection, ensuring improved lung tissue distribution, prolonged drug retention, and a favorable safety profile. Flexibility in administration routes and dosing regimens further highlights the clinical potential of EV-based therapies in respiratory oncology [42].

EV-based gene therapy represents another rapidly growing area of translational research. For example, a phase I clinical trial (NCT03608631) is currently investigating the use of engineered EVs to deliver specific genes to pancreatic cancers harboring KRAS G12D mutations (Fig. 1). By targeting EVs to the TME via intravenous administration, researchers aim to maximize on-target gene modulation while minimizing systemic exposure and off-target effects. Similarly, phase I and phase II clinical trials (NCT01159288) have evaluated the safety and feasibility of engineered dendritic cell–derived EVs (DEXs) to deliver Melanoma Antigen Gene (MAGE) antigens in patients with advanced non–small cell lung cancer (NSCLC) (Fig. 1). To date, the results are encouraging; multiple intravenous administrations of DEX have achieved efficient cancer targeting without causing significant adverse effects, suggesting a favourable therapeutic window [51, 52].

Macrophage-derived EVs constitute another class of therapeutic vesicles. These vesicles are distinguished by their surface protein composition, which facilitates preferential accumulation in cancer tissues. All tumors characterized by an acidic microenvironment are particularly prone to internalize small EVs (sEVs), a characteristic that has been exploited to improve the delivery of chemotherapeutic agents such as paclitaxel (Ptx). Using gentle sonication, researchers have successfully encapsulated Ptx within macrophage-derived EVs, resulting in improved drug stability and enhanced cytotoxicity against multidrug-resistant (MDR) cancer cells [53, 54]. Importantly, this method has demonstrated the ability to minimize off-target toxicity while maximizing therapeutic effects [44, 55]. Moreover, sEVs exhibit selective cytotoxicity toward malignant cells with minimal impact on normal tissues [56].

In addition to intravenous and aerosolized administration, the oral administration of EV-based therapies is also being actively investigated. For example, milk-derived sEVs have been loaded with Ptx and tested in preclinical models of human lung cancer. In these studies, the oral administration of sEVs resulted in greater cancer growth inhibition than did the conventional intravenous administration of Ptx [57]. This noninvasive approach could facilitate more frequent dosing, thus improving patient compliance and reducing the overall therapeutic burden.

Taken together, the versatility and adaptability of EVs underscore their significant therapeutic potential in oncology. As research in this area continues to advance in this field, EV-based therapies are expected to not only complement but also redefine existing cancer treatment paradigms.

## Exercise-derived EVs as a novel strategy in oncology and cardio-oncology

Emerging evidence has increasingly highlighted the critical role of exercise-derived EVs as crucial mediators of the beneficial effects of PA on cancer biology, with particular emphasis on breast and colon cancer [22, 58–60].

Recent multi-omics studies have provided a deeper mechanistic understanding of ExEVs by integrating proteomics, lipidomics, metabolomics, and small RNA sequencing. These analyses reveal that ExEVs are enriched with exercise-responsive proteins involved in metabolic regulation, stress adaptation, and immune signaling, as well as lipid mediators that modulate inflammatory pathways and membrane dynamics [19, 35, 61, 62]. Notably, small RNA-seq and profiling datasets have identified specific myo-miRs, including miR-486 and miR-181a, which regulate cell cycle progression, apoptosis, and immune cell activation, thereby influencing tumor metabolism and immune infiltration [25, 28, 63]. Lipidomic profiling further demonstrates the presence of bioactive lipids such as sphingolipids and phosphatidylserine, which contribute to EV uptake and signaling within the TME [19, 35]. Metabolomic analyses indicate that ExEVs carry metabolites linked to oxidative stress control and mitochondrial function, supporting systemic metabolic reprogramming during exercise [30, 31]. Collectively, these multi-omics datasets strengthen the hypothesis that ExEVs act as complex molecular packages capable of remodeling the TME through coordinated metabolic and immunological pathways.

**Fig. 2:**
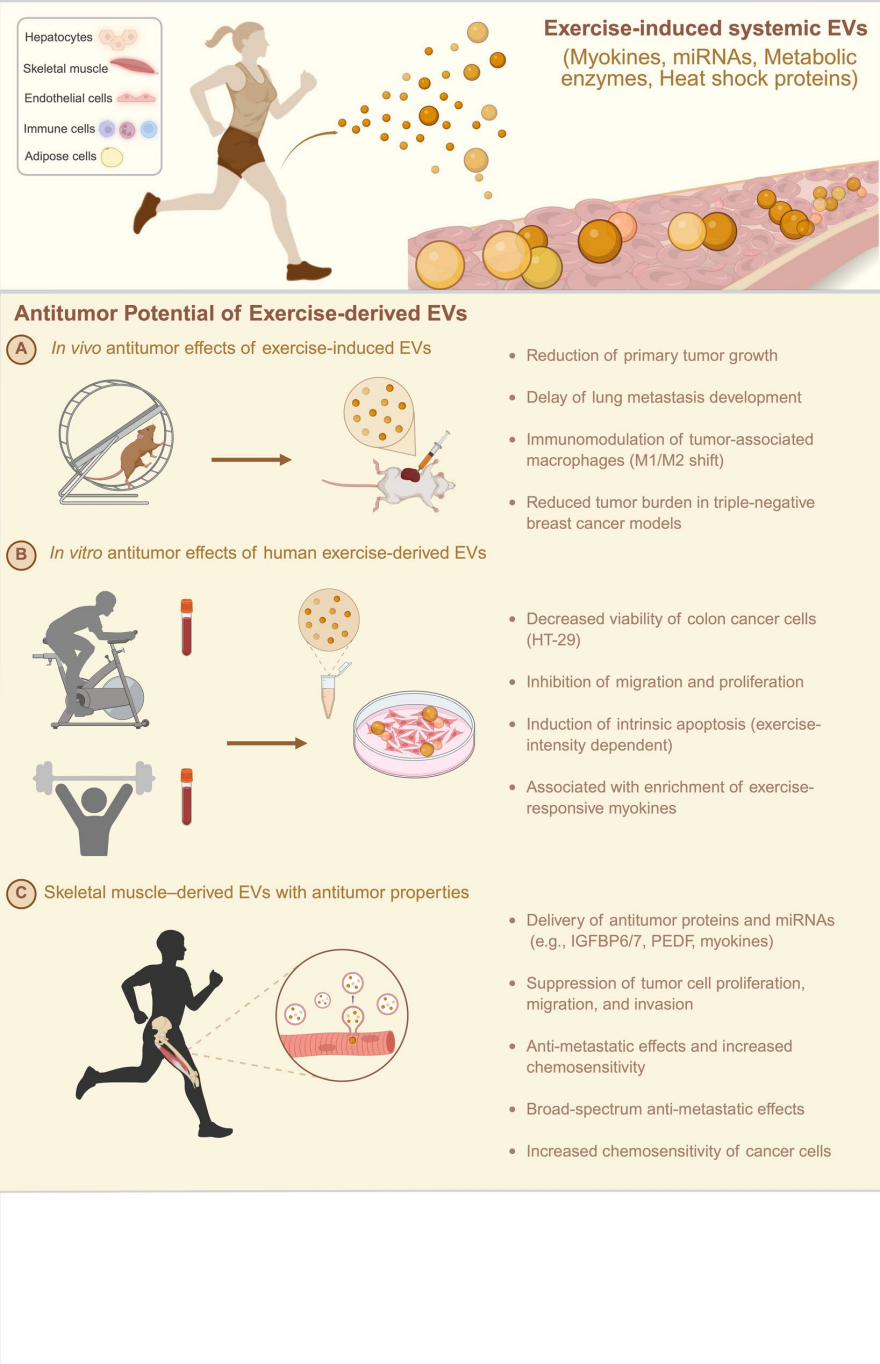
Exercise-derived extracellular vesicles (EVs) as mediators of antitumor effects. Schematic representation of systemic EVs released during physical activity from multiple tissues, including skeletal muscle, hepatocytes, endothelial cells, immune cells, and adipose tissue. These vesicles, enriched with myokines, microRNAs, metabolic enzymes, and heat shock proteins, enter the circulation and influence both tumor biology and cardiac function. (**A**) In vivo studies have shown that exercise-induced EVs reduce primary tumor growth, delay lung metastasis, modulate macrophage polarization (M1/M2 shift), and decrease the tumor burden in triple-negative breast cancer models. (**B**) In vitro experiments with human exercise-derived EVs demonstrated decreased viability of colon cancer cells (HT-29), inhibited migration and proliferation, and induced intrinsic apoptosis in an exercise-intensity–dependent manner. (**C**) Skeletal muscle–derived EVs deliver antitumor proteins and microRNAs (e.g., IGFBP6/7, PEDF, and myokines), suppress tumor proliferation, migration, and invasion, and exert broad-spectrum antimetastatic effects while increasing chemosensitivity

Preclinical studies have been crucial in uncovering how ExEVs function in cancer biology. Both human and animal models have shown that administering ExEVs can significantly reduce tumor growth and metastasis and improve the efficacy of chemotherapy, particularly in prostate and breast cancer models [58–60]. These EVs exert their anticancer effects by modulating key signalling pathways, altering the TME, and promoting a proinflammatory immune response (Table 2) (Fig. 2).

**Table 2:** Proposed mechanisms by which exercise-derived EVs target hallmarks of cancer

Hallmark of cancer	Experimental model	EV cargo changes	Cellular process	Evidence	References
*Proliferative/Growth* *Metastasis*	PC cells: PLS10 in F344 rats	*Cyp4b1*,* Notum*,* Pctp*, Dnajb5, Hspa5, Ltb4r2, Alox5 Zbtb1, *OXTR*,* DXO*	Metabolic processes; Cell survival Inflammation; T-cell function/development/ differentiation; Hormone signaling; RNA metabolism	↓ Cancer proliferation/growth ↓ Lung metastasis	Sadowska et al., 2022
*Proliferative/Growth*	PC cells (LNCaP, DU-145, and PC-3)	*IL-6*,* IL-15*,* FGF-21*, and *SPARC*	Metabolic processes; Immune function; Inflammation	↓ Cancer proliferation/growth	Kim et al., 2024
*Proliferative/Growth*	2 TNBC models: EO771 in C57BL/6 and 4T1 in BALB/c mice	NA	Immune function	↓ Cancer proliferation/growth ↓ Tumor burden TME remodelling	Mlynska et al., 2025
*Viability/Migration*	Colon cancer cells (HT-29)	NA	Cell survival	↓ Cancer cell viability ↓ Cancer cell migration Pro-apoptotic effect	Ozerklig et al., 2025

Alox5: arachidonate 5-lipoxygenase; Cyp4b1: cytochrome P450 family 4 subfamily B member 1; Dnajb5: DnaJ heat shock protein family (Hsp40) member B5; DXO: decapping exoribonuclease; EV(s): extracellular vesicle(s); ExEVs: exercise-derived extracellular vesicles; FGF-21: fibroblast growth factor 21; Hspa5: heat shock protein family A member 5 (GRP78/BiP); IL-15: interleukin-15; IL-6: interleukin-6; Ltb4r2: leukotriene B4 receptor 2; NA: not available/not assessed; OXTR: oxytocin receptor; PC: prostate cancer; Pctp: phosphatidylcholine transfer protein; SPARC: secreted protein acidic and rich in cysteine; TME: tumor microenvironment; TNBC: triple-negative breast cancer; Zbtb1: zinc finger and BTB domain–containing protein 1

Recent evidence further highlights that EVs, including ExEVs, orchestrate antitumor immunity by enhancing T-cell activation and NK-cell cytotoxicity [34, 36] and by increasing the antigen presentation and immunogenic cell death, thereby improving tumor visibility to the immune system [21, 35]. Importantly, multiple preclinical reports indicate that EV-based therapies can synergize with immune checkpoint blockade, including PD-1/PD-L1 and CTLA-4 inhibitors, by reducing immunosuppressive signaling and enhancing infiltration of cytotoxic lymphocytes into the tumor microenvironment [34, 36]. Together, these data position EVs as promising adjuncts to immunotherapy and reinforce the rationale for exercise-informed EV strategies in translational oncology.

The first study to demonstrate a direct tumor-suppressive effect of ExEVs was published in 2022, when Sadovska and colleagues reported that the administration of ExEVs to tumor-bearing rats resulted in an approximate 35% reduction in primary tumor size and appeared to delay the development of lung metastases [60]. Molecular analysis of the ExEV cargo revealed increased expression of genes encoding metabolic proteins, including Notum (palmitoleoyl-protein carboxylesterase), Pctp (phosphatidylcholine transfer protein), and Cyp4b1 (cytochrome P450 enzyme). Furthermore, the vesicles carried stress-related molecular chaperones such as Dnajb5 (a member of the Hsp40 family) and Hspa5 (a member of the Hsp70 family), both of which are essential for protein folding and cellular resilience under stress. The EVs also contained proinflammatory mediators such as Ltb4r2 (leukotriene B4 receptor 2) and Alox5 (arachidonate 5-lipoxygenase), along with molecules involved in immune cell differentiation (Zbtb1), hormone signaling (oxytocin receptor, OXTR), and RNA metabolism (DXO, a decapping exoribonuclease). Although the sample size was limited, these findings highlight the bioactive cargo of ExEVs and suggest a possible mechanistic role in suppressing tumor progression and metastatic spread in vivo.

Further, in 2024, Kim and colleagues compared the effects of the two main exercise modes, aerobic and resistance, on EV abundance and cargo content, and their uptake in PCa (prostate cancer) cells in patients with localised or advanced PCa [64]. They found that ExEV cargo was enriched in irisin, IL-6, IL-15, FGF-21, and SPARC, which form a core group of exercise-responsive myokines that mediate metabolic, immune, and anti-inflammatory effects. Since Kim and colleagues previously reported a reduced tumor growth after treating PCa cells with exercise-derived serum from similar patient populations, along with increased plasma levels of the same myokines later detected in EVs [65], it is reasonable to hypothesize that part of the anti-tumor effect may be mediated by the ExEV cargo.

In 2025, Mlynska and colleagues demonstrated that EVs derived from exercise-conditioned mice significantly delayed tumor growth, reduced the tumor burden, and remodeled the TME in 2 TNBC mouse models (mammary cancer cell lines 4T1 and EO771) through immunomodulation rather than direct cytotoxicity [58]. Specifically, ExEVs induced an immunosuppressive macrophage phenotype (CD206 upregulation and CD86 downregulation) in EO771 tumors, whereas in 4T1 tumors, they promoted a proinflammatory shift toward a more antitumoral M1-like phenotype (Table 2) (Fig. 2). However, the lack of a more in-depth biomolecular approach, including a detailed molecular characterization of the ExEV cargo, limits our understanding of the mechanisms driving immune TME reprogramming [58].

In the same year, Ozerklig and colleagues investigated for the first time the biological effects of human ExEVs on human colon cancer cells [59]. They reported that EVs isolated from the serum of healthy males subjected to high-intensity interval exercise reduced cell viability, inhibited migration and proliferation, and induced intrinsic apoptosis in HT-29 cells in an intensity-dependent manner (Table 2) (Fig. 2). However, this study also has several limitations. For example, ExEVs were tested only on the HT-29 cancer cell line and not on noncancerous cells, making it difficult to determine the specificity of the observed antitumor responses. Moreover, the study included only young, healthy male donors, which limits the generalizability of the findings across genders and age groups. The absence of a sedentary control group further limits the ability to isolate the specific contribution of exercise to the biological effects of EVs. Similar to the study by Mlynska and colleagues [58], this work also lacks a comprehensive biomolecular and mechanistic investigation to clarify how ExEVs mediate the observed cellular effects.

Several studies have demonstrated that skeletal muscle-derived EVs, particularly those from muscle progenitor cells, are enriched with proteins and miRNAs that can inhibit cancer cell proliferation, migration, and invasion [66–68] (Fig. 2). For instance, exosomes from skeletal muscle progenitor cells have been found to suppress prostate cancer cell growth by activating cell cycle inhibitors and pro-apoptotic genes, as well as by delivering anti-proliferative proteins such as IGFBP6/7 and pigment epithelium-derived factor (PEDF) [68]. Moreover, these EVs showed to reduce the migratory capacity of ovarian and pancreatic cancer cells, highlighting a potential broad-spectrum antimetastatic effect [61, 68].

Proteomic and transcriptomic analyses of muscle-derived EVs have revealed molecules that negatively regulate oncogenic signaling pathways, modulate the TME, and increase the cancer cells’ sensitivity to chemotherapy [19, 35, 61, 62]. These findings suggest that skeletal muscle-derived EVs are promising candidates for the delivery of bioactive molecules with anticancer properties, either as stand-alone agents or in combination with existing therapies.

Exercise has been shown to stimulate the release of more than 300 bioactive components from skeletal muscle-derived EVs, including myokines, miRNAs, metabolic enzymes, and heat shock proteins [19, 69]. Many of these molecules have been implicated in tumor-suppressive pathways and the regulation of key cancer hallmarks, such as uncontrolled proliferation, resistance to cell death, and metastasis [61, 70–73]. Among these, myokines such as irisin, SPARC, and IL-6 have been reported to exert direct or indirect antitumor effects [66, 74].

Despite these promising findings, direct evidence from preclinical cancer models investigating the role of skeletal muscle-derived EVs remains limited. Further in vivo studies are needed to delineate their mechanisms of action, biodistribution, and therapeutic efficacy, which are essential steps to fully exploit their potential in oncology. Table 2 summarizes the emerging mechanisms by which exercise-derived EVs target hallmarks of cancer.

**Fig. 3:**
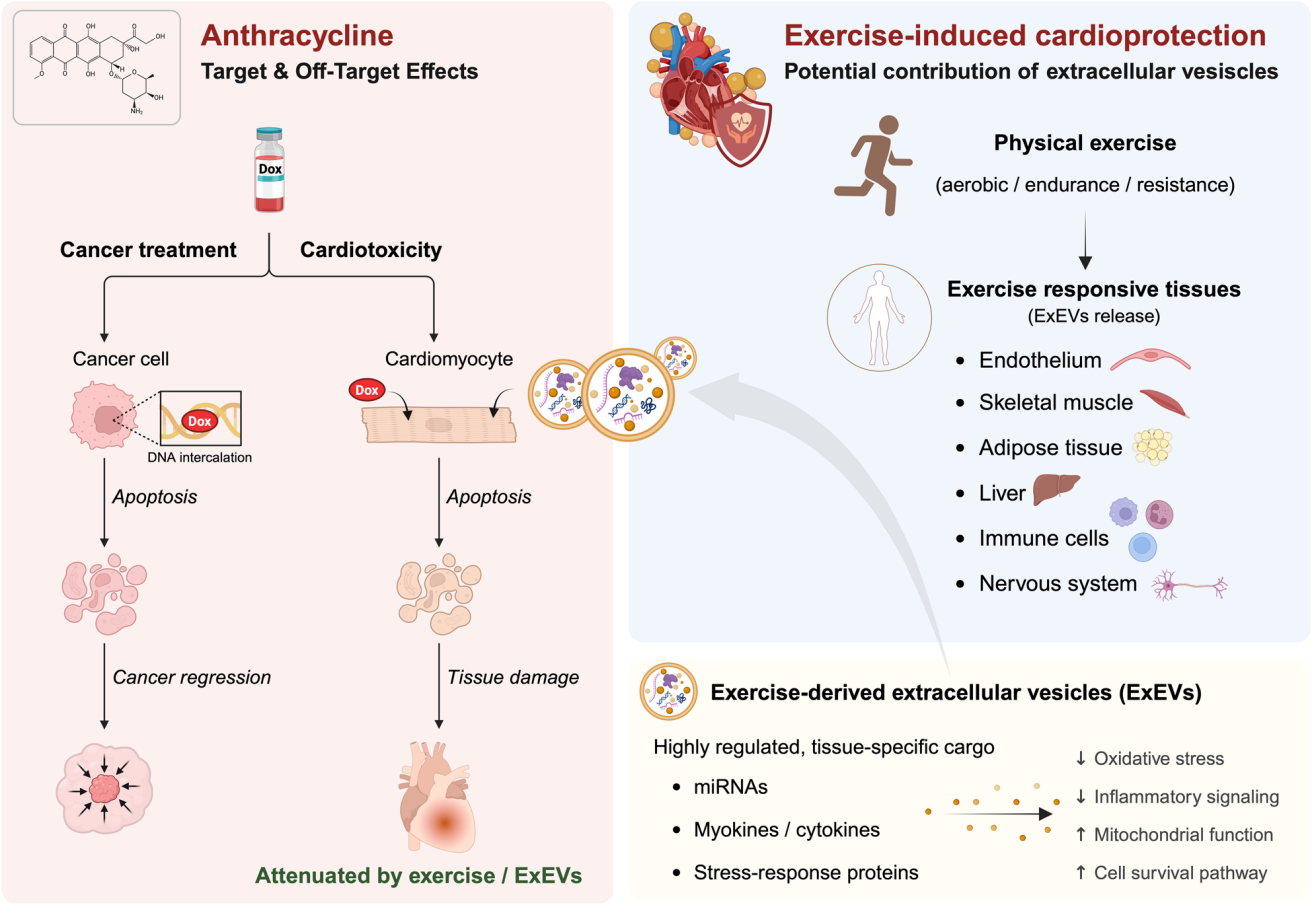
Exercise-induced cardioprotection and potential contribution of ExEVs in oncology. Schematic representation of anthracycline-associated cardiotoxicity and the potential involvement of ExEVs in exercise-induced cardioprotective responses. Anthracycline-based chemotherapy may induce cardiac injury through cardiomyocyte apoptosis, oxidative stress, inflammation, and mitochondrial dysfunction. PA activates a coordinated multi-organ response involving exercise-responsive tissues, including endothelium, skeletal muscle, adipose tissue, liver, immune cells, and the nervous system, promoting the release of ExEVs with highly regulated, tissue-specific molecular cargo. These EVs have been reported to modulate key pathways in cardiomyocytes, including oxidative stress, inflammatory signaling, mitochondrial function, and cell survival. EVs, extracellular vesicles; ExEVs, exercise-derived extracellular vesicles; PA, physical exercise; miRNAs, microRNAs

A growing area of interest concerns the cardioprotective effect of ExEVs in oncology patients, particularly those receiving anthracycline-based chemotherapy, which carries a well-documented, dose-dependent risk of cardiotoxicity [7, 75] (Fig. 3). Recent mechanistic evidence from cardiovascular research strengthens the hypothesis that ExEVs contribute directly to cardiac protection through their highly regulated, tissue-specific molecular cargo. During exercise, multiple tissues, including endothelial cells, skeletal muscle, adipose tissue, hepatocytes, immune cells, and neurons, show increased EV secretion and selectively load these vesicles with cardioprotective biomolecules [24] (Fig. 3).

From the vascular compartment, exercise stimulates endothelial cells to secrete EVs enriched in miR-342-5p, as demonstrated in mice undergoing four weeks of swimming and in athletes after one year of rowing. These EVs enhance Akt phosphorylation in cardiomyocytes by suppressing the apoptotic signaling mediated by caspase-9 and c-Jun N-terminal kinase 2 (JNK2), and by targeting protein phosphatase 1 F (PPM1F), thereby increasing cardiomyocyte survival and reducing susceptibility to ischemic injury [76]. Similarly, pre-conditioning mice with treadmill exercise prior to stroke accelerates recovery by promoting the release of endothelial progenitor cell–derived EVs, suggesting broader vasculoprotective effects [77].

Skeletal muscle represents another major source of ExEVs with cardioprotective potential [71, 78–80]. Acute exercise triggers the secretion of α-sarcoglycan–positive EVs enriched in miR-181a-5p [65], which suppress myocardial inflammation and oxidative stress, key drivers of cardiovascular disease [81]. Endurance training further enhances the release of exosomes containing HSP60 [84], a chaperone that boosts mitochondrial function in cardiomyocytes and confers resistance to cellular stress. Chronic swimming exercise has also been shown to increase muscle-derived EV production, improving glucose tolerance, reducing visceral adiposity, mitigating hepatic injury, and attenuating atherosclerosis in metabolically impaired mouse models [83]. Additionally, skeletal muscle–derived EVs enriched with FNDC5/irisin reduce vascular oxidative stress and inflammation, thereby slowing endothelial dysfunction and vascular aging [84, 85].

Adipose tissue also contributes to the cardioprotective ExEV pool [86]. Exercise promotes the browning of white adipose tissue (WAT) and expansion of brown adipose tissue (BAT), both of which shift adipose signaling toward a more cardioprotective profile [87]. In mice, four weeks of swimming training enhanced the release of BAT-derived EVs enriched in miR-125b-5p, miR-128-3p, and miR-30d-5p, which collectively inhibited the pro-apoptotic MAPK pathway in cardiomyocytes [86]. This led to a significant reduction in cell apoptosis and conferred protection against myocardial ischemia–reperfusion injury.

The liver, a central regulator of whole-body metabolism, is equally responsive to exercise and metabolic stress [88]. Under pathological conditions such as obesity or fatty liver disease, hepatocyte-derived EVs enriched in let-7b-5p inhibit mitochondrial oxidative phosphorylation and adipose browning, thereby exacerbating metabolic dysfunction and indirectly increasing cardiovascular strain [89–91]. Steatotic hepatocytes can also release EVs that disrupt coronary microvascular barrier integrity through the miR-7/LAMP1/Cathepsin B/NLRP3 axis, impairing coronary flow reserve [92]. Exercise counteracts these detrimental hepatic signals by promoting the release of EVs enriched in miR-122-5p, which target endothelial AGPAT1, enhancing fatty acid utilization and angiogenesis [93]. Consequently, liver-derived ExEVs operate at the intersection of metabolism and vascular regulation, reinforcing the multi-organ EV network that supports cardiovascular resilience.

Immune cells further integrate into this systemic response. Following cardiac injury, EVs released by T lymphocytes, macrophages, dendritic cells, and mast cells help to orchestrate inflammatory resolution and myocardial repair [94, 95]. Exercise enhances this immunomodulatory EV signaling: incremental cycling increases the release of leukocyte- and antigen presenting cells-derived EVs that improve vascular function and adaptive immunity [96]. Clinical studies show that exercise can reduce circulating pro-thrombotic microparticles from intermediate monocytes, alleviating vascular stress and contributing to cardioprotection in vulnerable populations such as renal transplant recipients [97]. These findings indicate that immune-derived ExEVs modulate inflammation, thrombosis, and vascular homeostasis in a manner that complements EV signals originating from endothelial, muscular, adipose, and hepatic tissues.

Importantly, emerging evidence highlights the nervous system as a contributor to ExEV-mediated cardioprotection [98]. Neuronal EVs are well established as mediators of intercellular communication within the central nervous system, regulating synaptic homeostasis and neuronal survival [99, 100]. Exosomes enriched in miR-124a released by neurons enhance astrocytic glutamate transporter expression, preventing excitotoxicity [101], while miR-150-3p–rich EVs from neural stem cells promote neuronal proliferation by inhibiting CASP2 signaling [102]. Beyond neuroprotection, neural stem cell–derived EVs delay ROS-induced mitochondrial permeability transition pore opening in cardiomyocytes via gp130/JAK/STAT signaling, reducing infarct size and protecting against oxidative injury [103]. Exercise further stimulates neuronal EV secretion enriched in BDNF, proBDNF, and humanin, linking neural adaptation to systemic protection and reinforcing neuro–cardiac communication pathways [104].

Despite ongoing efforts, the precise tissue-specific contributions remain incompletely defined. Nevertheless, the available evidence supports the concept that ExEVs function as an integrated, multi-organ communication system activated by exercise. By converging on pathways governing apoptosis, oxidative stress, inflammation, mitochondrial function, angiogenesis, vascular integrity, immune balance, and neuro–cardiovascular crosstalk, ExEVs provide a compelling mechanistic framework for exercise-induced cardioprotection [30, 31, 105, 106]. In the context of oncology, these vesicular signals may represent a key biological basis for mitigating anthracycline-induced cardiotoxicity and for developing novel ExEV-inspired therapeutic strategies (Fig. 3). Table 3 lists the main studies that have demonstrated an improvement in cardiovascular health induced by ExEVs.

**Table 3:** Proposed mechanisms by which exercise-derived EVs promotes cardiovascular health

Research subjects	EV cargo changes	Functional changes	References
Human	SOD3	↑ angiogenesis in endothelial cells	Abdelsaid et al., 2022
Human	MAP2K1	Protects cardiomyocytes from oxidative stress	Lisi et al., 2023
Human	miR-126-3p	Protects endothelial cells from hypoxic damage	D’Souza et al., 2018
Human	Nampt	↓ Myocardial cell apoptosis, ↑ survival	Chong et al., 2022
Human	CAT	↑ Protective effects on the heart	Kobayashi et al., 2021
Mouse	miR-126	↑ Recruitment of EPCs to the site of injury	Alehossein et al., 2022
Mouse	miR-445, miR-29b	Prevents myocardial fibrosis and uncoupling	Chaturvedi et al., 2015
Rat	miR-338	Protect brain microvascular endothelial cells	Huang et al., 2021
Mouse	miR-122-5p	↑ Endothelial cell fatty acid utilization, ↑ angiogenesis	Lou et al., 2022
Mouse	miR-27a	↓ ROS production	Chen et al., 2024
Mouse	miR-126	↓ Cell apoptosis	Wang et al., 2020
Rat	BDNF	Promotes the recovery of neurological function after stroke	Barcellos et al., 2020
Mouse	miR-125-5p, miR-128-3p, miR-30d-5p	Protects the heart from myocardial ischemia/reperfusion injury	Zhao et al., 2022
Rat	miR342-5p	Protects the heart against myocardial ischemia/reperfusion	Hou et al., 2019

SOD3, extracellular superoxide dismutase; MAP2K1, mitogen-activated protein kinase kinase 1; Nampt, nicotinamide phosphoribosyltransferase; CAT, catalase; BDNF, brain-derived neurotrophic factor; EPCs, endothelial progenitor cells; ROS, reactive oxygen species; N2a cell, mouse neuroblastoma N2a cells; ↑ increase, ↓ decrease

In summary, increasing evidence highlights the multifaceted translational importance of ExEVs in oncology. These vesicles serve not only as messengers that convey the systemic benefits of PA but also as active participants that modulate cancer biology, enhance immune surveillance, and protect healthy tissues from treatment-related damage.

However, despite these encouraging findings, the current ExEV research landscape presents significant limitations and inconsistencies. Most studies are preliminary, with small sample sizes and heterogeneous methodologies, including variability in exercise protocols, EV isolation techniques, and donor characteristics. Mechanistic evidence linking specific ExEV cargos (i.e., proteins, miRNAs) to antitumor effects remains largely correlative, and causal relationships are poorly defined. Furthermore, many investigations lack comprehensive biomolecular characterization and fail to assess off-target effects or specificity in noncancerous cells. These gaps underscore the need for rigorous multi-omics profiling, standardized protocols, and functional validation in physiologically relevant models.

## Barriers to the clinical translation of exercise-derived/inspired EVs in oncology

Preclinical studies suggest that EVs released during exercise may also exhibit anticancer and cytoprotective effects, suggesting their application as novel biotherapeutics or as models for synthetic delivery systems, so-called *exercise-inspired EVs*. However, despite the growing interest, significant barriers prevent the clinical translation of these vesicles. These challenges range from biological complexity, manufacturing limitations, and regulatory ambiguity, as well as ethical and logistical considerations [107–110].

One of the main challenges is the biological heterogeneity of ExEVs [107, 108, 110]. These vesicles originate from different cell types, including skeletal muscle, endothelial cells, immune cells, and platelets, and their content and function are extremely variable depending on exercise parameters such as type, intensity, and duration. Host factors such as age, sex, training status, and disease state also significantly influence EV yield and cargo. This inter- and intraindividual heterogeneity complicates the determination of a standardized EV product with uniform biological activity. Inadequate mechanistic understanding of which EV components (e.g., specific microRNAs, proteins, or lipids) mediate therapeutic effects further limits advances toward reproducible, defined therapies (see Table 4).

**Table 4:** Barriers to clinical translation of exercise-derived EVs

Barrier Type	Description	References
Biological Complexity	EVs are highly heterogeneous in size, cargo, and surface markers. The specific bioactive components responsible for therapeutic effects are not fully understood, and it remains unclear which EV subtypes are most relevant for therapy.	Estes et al., 2022 Lee et al., 2019 Fattore et al., 2023
Manufacturing Limitations	Isolation and purification methods (e.g., ultracentrifugation, chromatography) often yield mixed EV populations with variable purity. Scaling up production while maintaining biological activity and cargo integrity is challenging. Lack of standardized protocols leads to batch variability and limits reproducibility.	Silva et al., 2021 Liangsupree et al., 2021 Steć et al., 2024 Lee et al., 2019
Regulatory Ambiguity	There is no clear regulatory classification for EVs (biologics, cell-derived products, or advanced therapy medicinal products). Regulatory agencies have yet to provide specific guidelines, complicating safety, quality control, and clinical trial design.	Silva et al., 2021 Steć et al., 2024
Ethical and Logistical Considerations	Issues include donor consent, sourcing of starting material, and ensuring consistent quality. Logistical challenges also arise in storage, transport, and integration into clinical workflows.	Steć et al., 2024

From a technological and manufacturing perspective, current EV isolation and purification techniques are not compatible with clinical-grade production. Methods such as ultracentrifugation, size exclusion chromatography, or polymer precipitation produce material with a high contaminant contents and low concentration that are not scalable [111]. In response to these limitations, new methods such as tangential flow filtration, affinity purification, capillary zone electrophoresis, and microfluidic-based systems are being explored, although the technologies need to be optimized for large scale, Good Manufacturing Practice (GMP)-compliant production [112–115].

The lack of defined protocols for EV isolation, quantification, and quality control complicates interlaboratory reproducibility and limits regulatory acceptability. Furthermore, the long-term storage stability of EVs, even under transport conditions, is a concern, as their contents may aggregate or degrade under suboptimal conditions.

The issue of regulatory classification represents another significant obstacle. EVs exist in a gray area between drug delivery vehicles, biologics, and cell-derived therapies, and existing paradigms fail to define their status. Consequently, ExEVs represent a new class of biologically derived, donor-dependent vesicles, whose therapeutic use can raise complex regulatory challenges. The lack of clear guidelines from regulatory bodies continues to complicate the clinical application of these vesicles [116].

Targeting and safety pose additional concerns. Although EVs are generally poorly immunogenic, their biodistribution after systemic administration has not been fully characterized. Accidental deposition in nontarget tissues or unconventional biological reactions could raise safety concerns. Indeed, another critical barrier to clinical translation is the limited understanding of systemic biodistribution and tissue tropism of EVs following administration. Recent advances in in vivo imaging technologies provide essential tools to overcome this gap. Positron Emission Tomography (PET) enables quantitative, real-time tracking of EV biodistribution across organs, offering high sensitivity and translational relevance for dose optimization and safety assessment [44, 117]. Similarly, Near-Infrared II (NIR-II) imaging allows deep-tissue visualization with minimal background interference, improving spatial resolution in preclinical models and facilitating longitudinal studies [118, 119]. In parallel, bioluminescence imaging using luciferase-tagged EVs provides a highly sensitive approach for monitoring EV persistence, clearance, and targeting dynamics in vivo [43, 120]. These technologies are not only pivotal for defining pharmacokinetics and off-target effects but also represent a prerequisite for regulatory validation and the rational design of EV-inspired nanomedicine platforms. Integrating advanced imaging into preclinical workflows will accelerate the development of safe and effective ExEV-based therapies.

Furthermore, the use of EVs from patients or healthy donors derived from exercise introduces content heterogeneity that can impact efficacy and safety profiles.

The clinical and ethical limitations of EV collection from human donors also complicate translation. It is difficult to standardize the exercise conditions under which EVs are collected, achieve donor compliance, and logistically manage repeat sampling. Furthermore, interindividual variability means that EVs collected from one session or donor cannot be directly compared with those from another, compromising reproducibility. These limitations have spurred interest in ExEV mimetics: engineered artificial vesicles or nanoparticles capable of mimicking the payload and bioactivity of endogenous ExEVs. Although these platforms offer promise in terms of scalability and design, they are still in the early stages of development and require extensive testing [116].

Finally, the clinical translational landscape for ExEVs remains largely unexplored. To our knowledge, no human clinical studies have been conducted to evaluate the safety, biodistribution, or therapeutic efficacy of these vesicles. There are no standardized biomarkers for monitoring EV activity or target engagement in vivo, and optimal dosing regimens have not been defined. Without the completion of early-phase clinical trials, it will remain difficult to establish the therapeutic potential of these vesicles or define a regulatory pathway for approval.

In conclusion, although the concept of using exercise-derived or exercise-inspired EVs for therapeutic use is scientifically attractive, their clinical translation is limited by multiple interdependent challenges. Their advancement will require a concerted effort to standardize EV manufacturing and characterization methods, increase mechanistic understanding, clarify regulatory status, and design scalable deployment platforms. New EV engineering technologies, synthetic nanoparticle synthesis, and multiomics characterization are poised to overcome these challenges, but much remains to be done before ExEVs become a clinical reality.

## Extracellular vesicle-inspired mimetics: engineering exercise into nanoparticles

Given the biological complexity, heterogeneity, and translational challenges associated with native exercise-derived EVs, the development of EV-inspired synthetic mimetics has emerged as a rational strategy to translate exercise biology into clinically viable therapeutic platforms [39]. These engineered systems aim to reproduce key structural and functional properties of ExEVs, such as membrane composition, targeting behavior, and bioactive cargo, while overcoming donor dependence, batch variability, scalability constraints, and regulatory uncertainty [39, 115, 121].

Among EV-inspired platforms, lipid nanoparticles (LNPs) currently represent the most clinically advanced and scalable option. LNPs benefit from established GMP-compliant production pipelines and a well-defined regulatory framework, as demonstrated by their widespread clinical use for nucleic acid delivery [122]. By drawing inspiration from the surface markers and molecular cargo of exercise-derived EVs, LNPs can be engineered to encapsulate exercise-regulated miRNAs, proteins, or metabolites that recapitulate the antitumor and cardioprotective effects observed in preclinical exercise models. Conceptually, this includes myomiRs, such as miR-206 and miR-133, as well as additional exercise-responsive miRNAs implicated in cell-cycle regulation, apoptosis, immune modulation, and metabolic control [35, 123].

Preclinical studies provide a proof-of-concept for this approach. LNPs delivering tumor-suppressive miRNAs, including miR-199b-5p and miR-204-5p, have demonstrated robust antitumor activity in melanoma and glioblastoma models by targeting complementary oncogenic pathways [123–125]. These findings support the feasibility of using synthetic nanocarriers to emulate the biological activity of endogenous EV cargo, while offering superior control over composition, dosing, and biodistribution.

Importantly, recent advances in EV–LNP hybrid technologies further expand the translational potential of exercise-inspired nanomedicine. Hybrid systems combining natural EV membranes with synthetic LNP cores have been developed to enable the efficient co-delivery of mRNA or miRNA, while preserving EV-like targeting and biocompatibility [115, 121, 126]. In parallel, microfluidic-based EV–LNP fusion technologies have emerged as scalable and reproducible manufacturing strategies, allowing controlled vesicle fusion and cargo loading suitable for industrial production [115, 126]. These approaches directly address one of the main bottlenecks of native ExEVs, namely, scalability and batch-to-batch consistency, while retaining key biological features of EV-mediated communication.

Another rapidly evolving strategy involves cell-membrane–camouflaged nanoparticles, in which synthetic cores are coated with membranes enriched in EV-associated surface proteins such as CD63, HSP70, and other stress- or exercise-related markers [127, 128]. These biomimetic coatings enhance circulation time, immune evasion, and tissue targeting, and conceptually align with the idea of reproducing exercise-induced EV signatures without relying on donor-derived vesicles. Such platforms are particularly relevant for translating exercise-specific surface cues into programmable nanotherapeutics.

Beyond oncologic targeting, EV-inspired LNPs and hybrid systems also offer unique opportunities for combination therapies [40]. Nanoparticles can be co-loaded with chemotherapeutic agents (e.g., doxorubicin) and exercise-mimetic biomolecules to enhance antitumor efficacy while mitigating systemic toxicity [42]. Their modular design allows customization based on tumor subtype, disease stage, and patient-specific risk profiles, in line with precision oncology and cardio-oncology paradigms.

Notably, exercise-inspired nanovesicles may be especially valuable in cardio-oncology, where cardiotoxicity limits the long-term efficacy of anticancer treatments. By incorporating cardioprotective miRNAs, stress-response proteins, or metabolic regulators identified in ExEVs, EV-inspired nanoparticles could provide myocardial protection independently of physical activity itself. This represents a critical advantage for frail patients, individuals undergoing intensive chemotherapy, or those unable to participate in structured exercise programs.

Although still at an early stage, EV-inspired and EV–LNP hybrid nanotechnologies represent a crucial translational bridge between exercise biology and clinical nanomedicine [129]. Continued advances in nanoparticle engineering, microfluidic manufacturing, surface functionalization, and multi-omics–guided cargo selection are expected to further refine these platforms. Collectively, these developments position exercise-inspired nanomedicine within the broader and rapidly expanding field of EV–LNP hybrid therapeutics, offering a realistic and scalable pathway toward clinical implementation.

## Translational roadmap and policy outlook for exercise-derived EVs in oncology

Unlocking the clinical potential of exercise-derived or exercise-inspired EVs in oncology requires a strategic, phased, and interdisciplinary translational network. While native exercise-derived EVs (ExEVs) hold substantial biological promise as mediators of antitumor and cardioprotective effects, their inherent heterogeneity, low yield, donor dependence, and manufacturing constraints limit direct clinical applicability. Consequently, ExEVs should be viewed not only as candidate biotherapeutics, but also as biological blueprints informing the rational design of engineered, clinically tractable nanomedicine platforms [39, 129].

Given the inherent heterogeneity, low yield, and logistical challenges of producing native EVs, a promising avenue is the development of exercise-inspired lipid nanoparticles (LNPs) that mimic the molecular cargo and membrane characteristics of exercise-evoked EVs [40, 41]. These synthetic constructs aim to replicate the beneficial bioactivity of physiological EVs while enabling standardized production, targeted release, and greater therapeutic precision. Importantly, recent advances in EV–LNP hybrid systems, microfluidic-based vesicle fusion, and membrane-camouflaged nanoparticles further strengthen the feasibility of translating exercise biology into clinically deployable nanotherapeutics [115, 121, 126].

A critical prerequisite for advancing these platforms is the integration of high-resolution multi-omics datasets, including proteomics, small RNA sequencing, lipidomics, and metabolomics. Such datasets are essential for identifying the specific EV-associated molecules that mediate antitumor activity, immune modulation, and cardioprotection, and for guiding the rational selection of cargos to be incorporated into EV-inspired nanoparticles [33, 35]. Omics-driven design will be particularly important to avoid empiricism and to ensure mechanistic fidelity between exercise-derived signals and their synthetic mimetics.

Emerging computational approaches are critical enablers for next-generation exercise-inspired therapeutics. Artificial intelligence (AI) assisted tools for EV cargo prediction accelerate the identification of exercise-responsive miRNAs, proteins, and metabolites, guiding rational design of EV-based or EV-mimetic platforms [130, 131]. In parallel, systems biology modeling provides a framework to simulate complex physiological networks, including exercise-induced metabolic shifts and tumor–immune interactions, which are difficult to capture experimentally [132, 133]. Together, these integrative strategies enhance mechanistic understanding, support the precision engineering of EV-inspired nanoparticles, and enable in-silico optimization of therapeutic interventions. Incorporating these approaches into the translational roadmap will be essential to overcome current limitations and to advance personalized exercise-oncology paradigms.

To advance these platforms toward clinical translation, harmonizing methodologies for EV isolation, quantification, and functional validation is essential. The current MISEV guidelines provide a fundamental framework [134], but adaptations are needed to capture the unique characteristics of EVs produced under dynamic physiological conditions, such as acute or chronic exercise [16, 18]. We suggest the development of a dedicated quality and functionality standard: the Minimum Functional Information for Exercise EVs (MiFIEV). This framework should include both biophysical parameters (e.g., size, concentration, and tetraspanin profile) and functional assays (e.g., cancer cell viability, immune activation, and mitochondrial recovery) as mandatory parameters for EV characterization.

Contextualizing ExEVs within the broader EV landscape is essential to highlight their translational distinctiveness. While hypoxia- and fasting-induced EVs share overlapping stress-response pathways (e.g., heat shock proteins, metabolic regulators), exercise-derived EVs differ by orchestrating a coordinated, multi-organ endocrine response that simultaneously modulates metabolism, immunity, and vascular function. This systemic integration underpins their unique therapeutic potential and justifies the development of exercise-inspired EV platforms as a distinct class of interventions [19, 135, 136].

The translation of exercise- and EV-based therapies will depend on the integration of multiple disciplines: (1) Exercise physiology, to define optimal stimuli for therapeutic EV release; (2) Oncology, to identify clinical niches and tumor-specific vulnerabilities and clinically meaningful endpoints; (3) Bioengineering, to develop scalable, GMP-compliant delivery systems; (4) Immunology, to assess immune modulation and synergy with immunotherapies; and (5) Regulatory sciences, to clarify classification, safety requirements, and approval pathways.

To operationalize this approach, we suggest the creation of Exercise-Oncology Translational Consortia, which incorporate shared EV biobanks, standardized protocols, and multicenter clinical trial networks. This infrastructure will be critical to accelerating the discovery, validation, and cross-disease comparison, while ensuring reproducibility and regulatory alignment across institutions.

A structured translational roadmap should guide this process through four critical phases: discovery, design, validation, and implementation, as summarized in Table 5. This framework aligns biological discovery with technological readiness, regulatory feasibility, and clinical need, fostering the emergence of EV-based or EV-inspired adjunct therapies that enhance therapeutic efficacy while minimizing systemic toxicity.

**Table 5:** Stepwise roadmap for the clinical translation of exercise-derived and -inspired extracellular vesicles in oncology

Phase	Milestone	Key Activities	Challenges
I. Preclinical Discovery	Mechanistic validation	- Omics profiling of EV cargo - Functional bioassays (e.g., MiFIEV framework) - Tumor subtype–specific efficacy studies	Cargo heterogeneity; model standardization
II. Optimization & Engineering	Design of EV mimetics	- Selection of exercise-regulated biomolecules - Engineering of scalable LNP systems - Targeting and cargo loading strategies	Stability; delivery efficiency
III. Regulatory & Safety Alignment	GMP production and early trials	- Define regulatory classification (biologic vs. nanodrug) - Toxicology and pharmacokinetics studies - Early engagement with EMA/FDA	Regulatory ambiguity; safety concerns
IV. Clinical Implementation	First-in-human application	- Dose finding and safety trials (oncology and/or cardio-oncology) - Biomarker-guided patient selection - Long-term outcome tracking	Inter-patient variability; endpoint selection

EV, Extracellular Vesicle; MiFIEV, Minimal Functional Information for Exercise EVs; LNP, Lipid Nanoparticle; GMP, Good Manufacturing Practice; EMA, European Medicines Agency; FDA, Food and Drug Administration

Ultimately, the successful integration of exercise-derived or mimetic EVs into oncology will depend on precision-based strategies, including EV-to-tumor matching, in which the vesicular cargo is tailored to the molecular and immunological landscape of individual tumors. Such an approach could enable multitarget, low-toxicity interventions with broad applicability across cancer types, positioning exercise-inspired nanomedicine as a transformative component of personalized oncology and cardio-oncology.

## Policy and funding outlook

The successful translation of exercise-inspired EV therapies will also depend on alignment with funding priorities, regulatory frameworks, and policy agendas. It is encouraging to note that recent strategic initiatives by the European Commission (e.g., Horizon Europe Cancer Mission, EIC Pathfinder), the NIH (e.g., the Exercise Oncology Program), and national funding agencies reflect a growing interest in lifestyle-integrated biotechnologies and next-generation drug delivery systems (European Commission, 2023; NIH, 2024).

Furthermore, regulatory agencies are beginning to outline clearer pathways for EV-based interventions. The European Medicines Agency (EMA) published a discussion paper in 2025 addressing the classification and quality control of EV-based therapies [137], whereas the U.S. Food and Drug Administration (FDA) has published preliminary guidelines on cell-derived nanoparticles [138, 139].

The recent publication titled *“Guidance on the clinical application of extracellular vesicles”* by the Japanese Society for Regenerative Medicine and the Japanese Society for Extracellular Vesicles [140] marks a significant step forward in the clinical translation of EV-based therapies. This document outlines three key pillars: (1) Risk assessment, including source cell safety, biological contaminants, and viral transmission risks; (2) Manufacturing process control, emphasizing GMP compliance, raw material traceability, and scalable purification technologies; and (3) Clinical efficacy validation, requiring standardized EV markers, functional assays, and inter-batch consistency. Table 6 provides a cross-disciplinary synthesis of the key domains, strategies, and stakeholders needed to enable full translational impact.

**Table 6:** Functional and strategic dimensions of exercise-derived EV translation

Domain	Objective	Strategic Actions
Mechanistic Biology	Define antitumor and cardioprotective functions	- EV profiling post-exercise - In vitro co-culture and in vivo modeling
Biotechnology	Engineer exercise-mimetic LNPs with enhanced delivery	- Synthetic EV surface design - Cargo encapsulation techniques
Immuno-oncology	Enhance immune activation and tumor microenvironment control	- EV modulation of TILs and cytokine profiles - Synergy with ICB therapies
Regulatory Science	Enable clinical approval and manufacturing scale-up	- MISEV adaptation for MiFIEV - GMP guidelines and quality control pipelines
Health Policy & Funding	Align funding with translational priorities	- Calls referencing EVs and exercise oncology - Interdisciplinary grant consortia

EV, Extracellular Vesicle; LNP, Lipid Nanoparticle; TILs, Tumor-Infiltrating Lymphocytes; ICB, Immune Checkpoint Blockade; MISEV, Minimal Information for Studies of EVs; MiFIEV, Minimal Functional Information for Exercise EVs; GMP, Good Manufacturing Practice; EMA, European Medicines Agency; FDA, Food and Drug Administration; NIH, National Institutes of Health

These recommendations, which are currently undergoing global discussion form a critical foundation for ensuring the safety, quality, and reproducibility of therapeutic EVs. Harmonizing such frameworks across international regulatory environments will be essential to accelerate the clinical deployment of both natural and engineered EV platforms, including those derived from or inspired by exercise.

## Conclusion

Exercise-derived extracellular vesicles represent a promising and largely unexplored frontier in cancer therapy. Preclinical studies consistently demonstrate that these vesicles carry a complex molecular cargo capable of modulating tumor progression, enhancing immune surveillance, and protecting healthy tissues from chemotherapy-induced damage. Recent multi-omics datasets provide a mechanistic foundation for these effects, revealing exercise-responsive proteins, metabolites, and myo-miRs that influence tumor metabolism and immune infiltration.

Despite this potential, clinical translation remains a challenging endeavor. Bridging the gap between the laboratory and the bedside requires the integration of advanced in vivo imaging technologies (e.g., PET, NIR-II, luciferase-tagged EVs) to define biodistribution and safety, alongside standardized manufacturing protocols and regulatory frameworks. Furthermore, synergy between EV-based strategies and immune checkpoint blockade, as well as the development of EV-inspired mimetic nanoparticles, offers a realistic pathway to scalable and personalized interventions.

By combining mechanistic insights, engineering innovation, and interdisciplinary collaboration, exercise-informed nanomedicine can evolve from concept to clinical reality. These innovations align with the goals of precision oncology, offering new strategies to improve outcomes and minimize toxicity. The time has come to transform the molecular benefits of exercise into actionable therapies, what is now a scientific curiosity must become a translational priority.

## Data Availability

Not applicable.

## Abbreviations

AI: Artificial Intelligence
AICAR: 5-Aminoimidazole-4-carboxamide ribonucleotide
AMPK: 5′ Adenosine Monophosphate–Activated Protein Kinase
BAT: Brown Adipose Tissue
BDNF: Brain-Derived Neurotrophic Factor
CRISPR: Clustered Regularly Interspaced Short Palindromic Repeats
CTLA-4: Cytotoxic T-Lymphocyte–Associated Protein 4
DEX(s): Dendritic Cell–Derived Extracellular Vesicle(s)
Dox: Doxorubicin
EMA: European Medicines Agency
ERR: Estrogen-Related Receptor
EV(s): Extracellular Vesicle(s)
ExEV(s): Exercise-Derived Extracellular Vesicle(s)
FDA: Food and Drug Administration
FNDC5: Fibronectin Type III Domain Containing 5
GMP: Good Manufacturing Practice
Hsp: Heat Shock Protein
IGFBP: Insulin-like Growth Factor–Binding Protein
IL-6: Interleukin-6
JNK2: c-Jun N-terminal Kinase 2
LNP(s): Lipid Nanoparticle(s)
MAPK: Mitogen-Activated Protein Kinase
M1/M2: Macrophage Polarization States (Pro- vs. Anti-inflammatory)
MAGE: Melanoma Antigen Gene
MiFIEV: Minimum Functional Information for Exercise EVs
MISEV: Minimal Information for Studies of Extracellular Vesicles
miRNA(s): MicroRNA(s)
NCT: National Clinical Trial (identifier for clinical studies)
NIR-II: Near-Infrared II
NK: Natural Killer (cells)
NSCLC: Non–Small Cell Lung Cancer
OXTR: Oxytocin Receptor
PA: Physical Activity
PCa: Prostate Cancer
PD-1: Programmed Death-1
PEDF: Pigment Epithelium–Derived Factor
PPARδ: Peroxisome Proliferator-Activated Receptor Delta
Ptx: Paclitaxel
ROS: Reactive Oxygen Species
sEV(s): Small Extracellular Vesicle(s)
SPARC: Secreted Protein Acidic and Rich in Cysteine
TME: Tumor Microenvironment
TNBC: Triple-Negative Breast Cancer
WAT: White Adipose Tissue
